# Aerosol Inhalation of Heat-Killed *Clostridium butyricum* CGMCC0313-1 Alleviates Allergic Airway Inflammation in Mice

**DOI:** 10.1155/2022/8447603

**Published:** 2022-08-05

**Authors:** Laodong Li, Qixiang Sun, Huan Xiao, Qiannan Zhang, Siyue Xu, Lejin Lai, Zhengning Li, Chaoqian Li

**Affiliations:** ^1^Pulmonary and Critical Care Medicine Ward, The First Affiliated Hospital of Guangxi Medical University, Nanning, 530021 Guangxi, China; ^2^Pulmonary and Critical Care Medicine Ward, The Fourth Affiliated Hospital of Guangxi Medical University, Liuzhou, 545005 Guangxi, China; ^3^Department of Emergency, The First Affiliated Hospital of Guangxi Medical University, Nanning, 530021 Guangxi, China

## Abstract

Epidemiological studies have shown that exposure to beneficial microorganisms can reduce the risk of asthma, but the clinical use of live probiotics is controversial due to the risk of infection. As heat-killed probiotics can also exhibit immunomodulatory activity, this study is aimed at investigating whether heat-killed *Clostridium butyricum* (HKCB) CGMCC0313-1 could reduce allergic airway inflammation in an ovalbumin-induced mouse model. Mice received aerosol inhalation of HKCB, oral administration of HKCB, or oral administration of live *Clostridium butyricum* (CB) during sensitization. Bronchoalveolar lavage fluid cell number, histology, and levels of the cytokines interferon-gamma and IL-4, the autophagy-related proteins LC3B, Beclin1, and p62, and members of the nuclear factor kappa B (NF-*κ*B)/NLRP3 inflammasome signaling pathway were examined. Our results demonstrated that aerosol inhalation of HKCB, oral HKCB administration, and oral live CB administration alleviated allergic airway inflammation and mucus secretion in allergic mice. Aerosol inhalation of HKCB was the most effective method; it restored the Th1/Th2 balance, ameliorated autophagy, and inhibited the NF-*κ*B/NLRP3 inflammasome signaling pathway in the lungs of allergic mice. Thus, aerosol inhalation of HKCB could be a promising strategy for the prevention or treatment of asthma.

## 1. Introduction

Asthma is a common chronic respiratory disease characterized by airway inflammation, goblet cell metaplasia, mucus hypersecretion, and, ultimately, airway remodeling. More than 350 million individuals suffer from asthma globally, and its prevalence continues to increase in some countries [1, 2]. Although asthma is a heterogeneous disease, allergic airway inflammation plays an important role in the pathogenesis of most asthma cases [3].

Epidemiological studies have demonstrated that exposure to an environment rich in beneficial microorganisms can reduce the risk of asthma [4, 5]. These protective microorganisms may regulate the immune system through the respiratory tract or intestine, providing protection against asthma [4, 6]. Probiotics are a set of living nonpathogenic microorganisms that confer beneficial effects on the health of the host when administered in adequate quantities [7]. Several animal studies have shown a clear preventive and therapeutic effect of probiotics against asthma, but so far, clinical trials have shown ambiguous results [6, 7]. Recently, a meta-analysis of randomized controlled trials indicated that orally administration probiotics, such as *Lactobacilli* or *Bifidobacteria*, did not prevent asthma in infants [8]. Moreover, safety issues associated with the use of live microorganisms remain a matter of debate.

Similar to probiotics, heat-killed probiotics also have anti-inflammatory and immunomodulatory activities, which has led to increased interest in using heat-killed probiotics to prevent and treat diseases [9]. A growing body of evidence has shown that nasal or oral administration of heat-killed probiotics can reduce allergic airway inflammation and might be a promising approach for the prevention and treatment of asthma [10, 11]. Recent studies have indicated that *Clostridium* may be an important microorganism against allergic diseases [5, 12]. *Clostridium butyricum* (CB) CGMCC0313-1 is a member of *Clostridium* cluster I and is used as a probiotic in clinical practice [13]. Previous studies have confirmed that oral administration of CB CGMCC0313-1 can inhibit allergic inflammation [14, 15]. However, whether heat-killed CB (HKCB) CGMCC0313-1 would have similar beneficial effects remains unknown. This study is aimed at comparing the effects of aerosol inhalation of HKCB, oral HKCB administration, or oral live CB administration in reducing allergic airway inflammation in mice and investigating the underlying mechanism of the most effective method.

This study concluded that while aerosol inhalation of HKCB, oral HKCB administration, and oral live CB administration alleviated the allergic airway inflammation in mice, aerosol inhalation of HKCB was the most effective in this regard. It, thus, may serve as an effective strategy for the prevention or treatment of asthma.

## 2. Materials and Methods

### 2.1. Preparation of CB and HKCB

CB CGMCC0313-1 powder (Kexing Biotech Company, Shandong, China) was stored at 4°C and prepared by suspending in sterile phosphate-buffered saline (PBS) at an approximate cell density of 5 × 10^7^ CFU/ml or 5 × 10^8^ CFU/ml. HKCB was obtained by steam sterilization (121°C and 110 kPa, 20 min) and stored at –20°C until use.

### 2.2. Animals

Male BALB/c mice aged 4–5 weeks were purchased from Changsha Tianqin Biotechnology Co., Ltd (Changsha, China) and acclimated to a new environment for 1 week. The mice were housed under specific pathogen-free conditions (23 ± 3°C) on a 12/12 h day/night schedule. Food and water were provided *ad libitum.* The experimental procedures strictly followed the Guide for the Care and Use of Laboratory Animals issued by the Ministry of Science and Technology of the People's Republic of China and were approved by the Ethics Committee of the Guangxi Medical University (Approval No. 202007060).

### 2.3. Study Design

Mice were randomly divided into eight groups (*n* = 8 mice/group): normal control (NC) group, asthma control group (OVA), aerosol inhalation of low-dose HKCB group (OVA+a.i. HKCB-L), aerosol inhalation of high-dose HKCB group (OVA+a.i. HKCB-H), oral low-dose HKCB group (OVA+oral HKCB-L), oral high-dose HKCB group (OVA+oral HKCB-H), oral low-dose live CB group (OVA+oral CB-L), and oral high-dose live CB group (OVA+oral CB-H).

**Figure 1:**
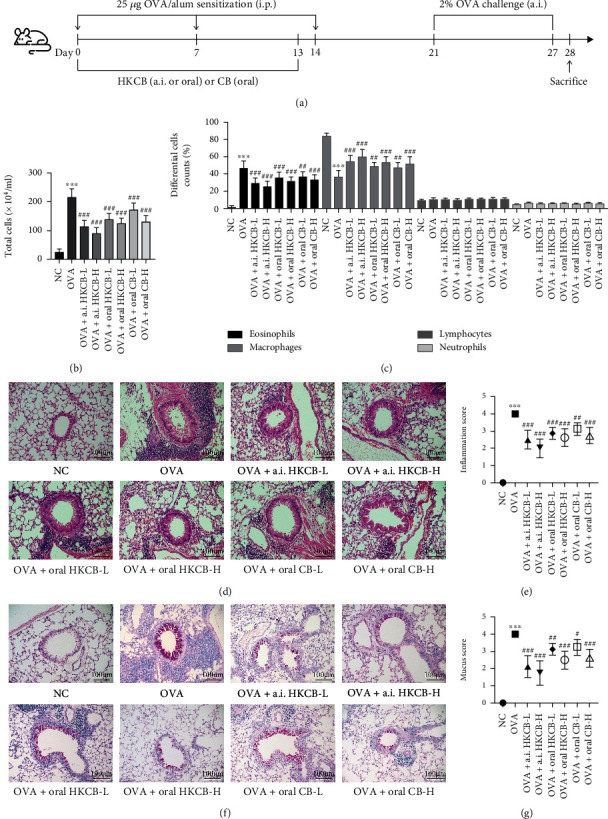
Aerosol inhalation of HKCB, oral HKCB administration, or oral CB administration alleviated allergic airway inflammation and mucus hypersecretion. (a) Experimental protocol. (b) Total inflammatory cell count in the BALF. (c) The percentages of differential inflammatory cells in the BALF. (d) HE staining of the lung tissues (magnification 200x). (e) Inflammation scores. (f) PAS staining of the lung tissues (magnification 200x). (g) Mucus scores. ^∗∗∗^*P* < 0.001 versus the NC group; ^#^*P* < 0.05, ^##^*P* < 0.01, and ^###^*P* < 0.001 versus the OVA group. i.p.: intraperitoneally; a.i.: aerosol inhalation; CB: *Clostridium butyricum*; HKCB: heat-killed CB; HE: hematoxylin-eosin; PAS: periodic acid-Schiff; BALF: bronchoalveolar lavage fluid.

Induction of allergic airway inflammation was performed as previously described [16], with slight modifications. With the exception of the NC group, all groups were intraperitoneally injected with 25 *μ*g OVA (Grade V, Sigma, St Louis, MO, USA) and 1 mg aluminum hydroxide (Meilunbio, Dalian, China) in 0.2 ml PBS on days 0, 7, and 14 for sensitization. On days 21–27, the mice were challenged by aerosol inhalation with 2% OVA in a closed chamber (30 × 20 × 30 cm, length, width, and height, respectively) for 30 min per day. Additionally, the model mice received either HKCB (1 × 10^7^ or 1 × 10^8^ CFU suspended in 20 ml PBS, 30 min per day) by aerosol inhalation, 0.2 ml HKCB (1 × 10^7^ or 1 × 10^8^ CFU/mouse per day) by intragastric administration, or 0.2 ml CB (1 × 10^7^ or 1 × 10^8^ CFU/mouse per day) by intragastric administration on days 0-13 for intervention. Mice in the NC group were sensitized and challenged with an equivalent dose of PBS (Figure 1(a)).

### 2.4. Bronchoalveolar Lavage Fluid (BALF)

The mice were sacrificed within 24 h of the last OVA challenge, followed by a tracheal cannula. For BALF collection, the trachea and right lungs were lavaged three times with 0.3 ml ice-cold PBS. The BALF was centrifuged at 1000 rpm for 10 min at 4°C, and the supernatant was stored at –80°C for further cytokine analysis. The sedimented cells were suspended in PBS for total inflammatory cell count and stained with Wright-Giemsa (Solarbio, Beijing, China) for differential cell counts. A total of 200 cells were counted on each slide.

### 2.5. Histopathology

The left lung lobes were fixed with 4% paraformaldehyde for 24 h at room temperature and embedded in paraffin. Sections (3–5 *μ*m) were stained with hematoxylin-eosin (HE) and periodic acid-Schiff (PAS) to evaluate airway inflammation, goblet cell hyperplasia, and mucus secretion using light microscopy. The severity of airway inflammation was scored semiquantitatively in a blind manner as follows: 0, no inflammatory cells; 1, few inflammatory cells; 2, a ring of inflammatory cells, a cell layer deep; 3, a ring of inflammatory cells, 2–4 cells deep; and 4, a ring of inflammatory cells > 4 cells deep [17]. The extent of goblet cell hyperplasia and mucus production was scored semiquantitatively in a blind manner according to the percentage of goblet cells in the epithelium as follows (internal diameter of airways: 100–200 *μ*m): 0, no goblet cells; 1, <25%; 2, 25-50%; 3, 51-75%, and 4, >75% [18].

### 2.6. Measurement of Cytokines in the BALF

The levels of interferon-gamma (IFN-*γ*) and IL-4 in the BALF were measured using ELISA kits (Cusabio Biotech, Wuhan, China) according to the manufacturer's protocols.

### 2.7. Immunohistochemistry

Antigen retrieval was performed on paraffin sections (3–5 *μ*m) by boiling in citrate antigen retrieval buffer (pH 6.0) or EDTA buffer (pH 8.0), followed by blocking in 3% H_2_O_2_ for 15 min. The sections were then incubated with primary antibodies against LC3B (Abmart, Shanghai, China), Beclin1 (Abmart), p62 (Wanleibio, Shenyang, China), nuclear factor kappa B (NF-*κ*B) p65 (Abmart), or NLRP3 (Wanleibio) at 37°C for 1 h and subsequently incubated with secondary antibodies (PV-9001, ZSGB-BIO, Beijing, China) according to the manufacturer's instructions. The expression levels of related proteins were scored semiquantitatively in a blind manner as the product of intensity scores (0, negative; 1, weak; 2, moderate; and 3, strong) and diffusion scores (1, 0-25% positive cells; 2, 26-50% positive cells; 3, 51-75% positive cells; and 4, >75% positive cells) [19].

### 2.8. Western Blotting

Total protein was extracted from the right lung tissues using RIPA buffer (Meilunbio, Dalian, China). Proteins were separated by 10% or 12% SDS-PAGE and then transferred to a polyvinylidene difluoride membrane (Millipore, Billerica, USA), which was blocked with 5% fat-free milk in TBST solution for 1 h at room temperature. The membranes were incubated with primary antibodies against LC3B (Abmart), Beclin1 (Abmart), p62 (Abmart), NF-*κ*B p65 (Abmart), NLRP3 (Wanleibio), ASC (Cell Signaling Technology, Inc. MA, USA), pro-caspase1 (Abcam, Cambridge, UK), caspase1 p20 (Wanleibio), and mature-IL-1*β* (Wanleibio) at 4°C overnight. The membranes were washed with TBST and incubated with a secondary antibody (SA5-35571, Thermo Fisher, USA) at room temperature for 70 min. Immunoreactive images were detected using the Odyssey system (LI-COR Biosciences, USA) and analyzed using the ImageJ software.

### 2.9. Statistical Analysis

Data were analyzed using the SPSS 25.0 software and expressed as mean ± standard deviation (SD). One-way analysis of variance (ANOVA) followed by least significant difference (LSD) analysis was used for between-group comparisons of data with a normal distribution and homogeneity of variance. The Mann-Whitney *U* test was used for data that did not have a normal distribution. Statistical significance was set at *p* < 0.05.

## 3. Results

### 3.1. Aerosol Inhalation of HKCB, Oral HKCB Administration, or Oral CB Administration Reduced Inflammatory Cell Abundance and Eosinophil Recruitment in the BALF

Compared with that of the NC group, the OVA group showed a significantly higher number of total inflammatory cells and a higher proportion of eosinophils in the BALF (*P* < 0.001). Aerosol inhalation of HKCB, oral HKCB administration, or oral CB administration significantly decreased the total inflammatory cell number and proportion of eosinophil (*P* < 0.01); these effects were the most prominent after the aerosol inhalation of high-dose HKCB (Figures 1(b) and 1(c)).

### 3.2. Aerosol Inhalation of HKCB, Oral HKCB Administration, or Oral CB Administration Alleviated Inflammatory Cell Infiltration and Mucus Hypersecretion

We further evaluated inflammatory cell infiltration, goblet cell hyperplasia, and mucus production in the lung tissues by HE staining and PAS staining. As shown in Figures 1(d)–1(g), there were significantly increased levels of inflammatory cells, goblet cells, and mucus surrounding the airways in the OVA group compared with that of the NC group (*P* < 0.001). However, inflammatory cell infiltration, goblet cell hyperplasia, and mucus hypersecretion in the lung tissues of mice subjected to the aerosol inhalation of HKCB, oral HKCB administration, and oral CB administration decreased significantly compared to of the case for the mice from the OVA group (*P* < 0.05). According to the results of total inflammatory cell and eosinophil counts in the BALF, HE staining, and PAS staining, aerosol inhalation of HKCB was the most effective method for alleviating allergic airway inflammation and was therefore selected for subsequent study.

### 3.3. Aerosol Inhalation of HKCB Restored the Th1/Th2 Balance

**Figure 2:**
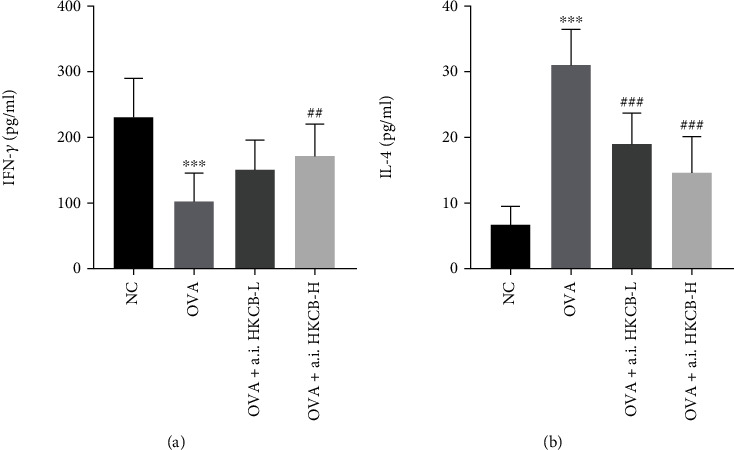
The effect of aerosol inhalation of HKCB on the cytokine concentrations in the BALF of allergic mice. (a) The concentration of IFN-*γ*. (b) The concentration of IL-4. ^∗∗∗^*P* < 0.001 versus the NC group; ^##^*P* < 0.01 and ^###^*P* < 0.001 versus the OVA group. a.i.: aerosol inhalation; CB: Clostridium butyricum; HKCB: heat-killed CB; BALF: bronchoalveolar lavage fluid.

The Th1/Th2 imbalance plays a critical role in the pathogenesis of allergic inflammation [20]. Therefore, we examined the effect of aerosol inhalation of HKCB on the levels of the Th1 cytokine IFN-*γ* and the Th2 cytokine IL-4 in the BALF. Compared to that of the NC group, the level of IFN-*γ* in the OVA group was significantly decreased (*P* < 0.001), but the level of IL-4 was significantly increased (*P* < 0.001). However, aerosol inhalation of high-dose HKCB significantly increased the level of IFN-*γ* (*P* < 0.01) and decreased the level of IL-4 (*P* < 0.001) compared to that of the OVA group. Aerosol inhalation of low-dose HKCB also significantly decreased IL-4 levels compared to that of the OVA group (*P* < 0.001) (Figure 2).

### 3.4. Aerosol Inhalation of HKCB Ameliorated Autophagy in the Lungs of Allergic Mice

**Figure 3:**
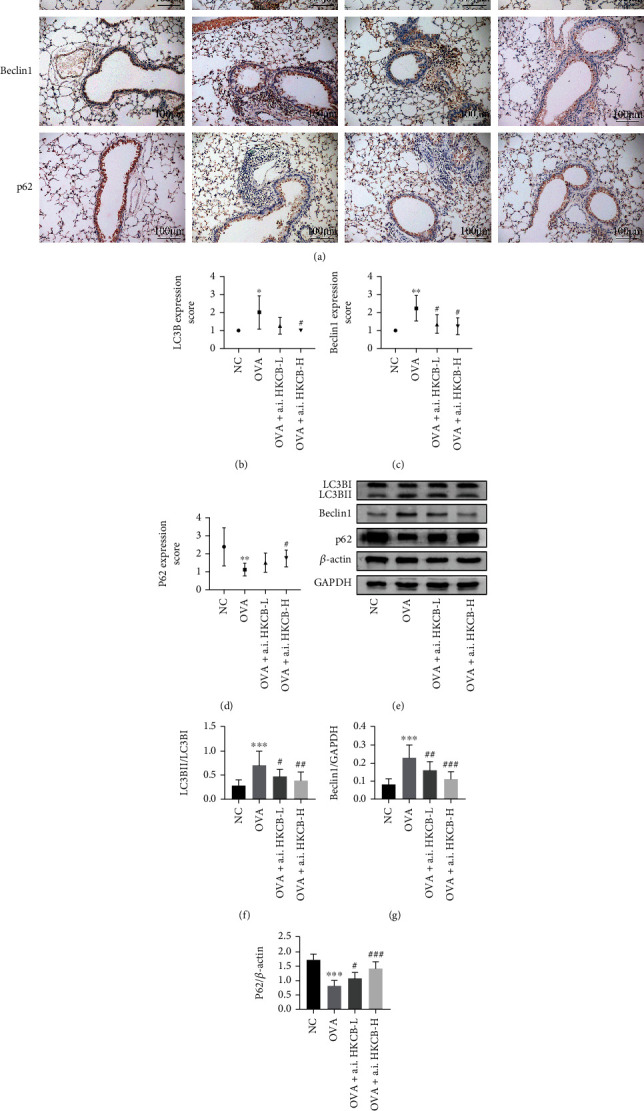
The effect of aerosol inhalation of HKCB on autophagy in the lung tissues of allergic mice. (a) Expression levels of LC3B, Beclin1, and p62 in lung tissues, as examined by immunohistochemistry staining (magnification 200x). (b–d) Expression scores of LC3B, Beclin1, and p62 in lung tissues. (e) Expression levels of LC3B, Beclin1, and p62 in lung tissues were examined using western blotting. (f–h) Relative expression levels of LC3BII/LC3BI, Beclin1, and p62 in lung tissues. ^∗^*P* < 0.05, ^∗∗^*P* < 0.01, and ^∗∗∗^*P* < 0.001 versus the NC group; ^#^*P* < 0.05, ^##^*P* < 0.01, and ^###^*P* < 0.001 versus the OVA group. a.i.: aerosol inhalation; CB: *Clostridium butyricum*; HKCB: heat-killed CB.

Abnormal autophagy is the pathological manifestation of allergic inflammation [21]. To investigate the effect of aerosol inhalation of HKCB on autophagy in the lung tissues of allergic mice, we used immunohistochemical staining to assess the expression of the autophagy-related proteins LC3B, Beclin1, and p62. We found that the expression of LC3B and Beclin1 in bronchial epithelial cells and airway inflammatory cells of the OVA group increased significantly (*P* < 0.05), while the expression of p62 in these cells decreased significantly compared to that in the NC group (*P* < 0.01). Enhanced autophagy of bronchial epithelial cells and airway inflammatory cells has been suggested to be involved in the pathogenesis of allergic asthma. However, aerosol inhalation of HKCB decreased the expression of LC3B and Beclin1 and increased the expression of p62 (Figures 3(a)–3(d) and Supplementary Figure S1).

We further evaluated the differences in the expression of these autophagy-related proteins in lung tissues using western blotting. The results demonstrated that the expression of LC3BII/LC3BI and Beclin1 were significantly higher (*P* < 0.001), whereas the expression of p62 was significantly lower (*P* < 0.001) in the OVA group than that in the NC group. Notably, aerosol inhalation of HKCB significantly decreased the expression of LC3BII/LC3BI and Beclin1 (*P* < 0.05) and significantly increased the expression of p62 (*P* < 0.05) compared to that of the OVA group (Figures 3(e)–3(h)). Taken together, these results suggest that aerosol inhalation of HKCB ameliorates autophagy in the lungs of allergic mice.

### 3.5. Aerosol Inhalation of HKCB Inhibited the NF-*κ*B/NLRP3 Inflammasome Signaling Pathway in the Lungs of Allergic Mice

**Figure 4:**
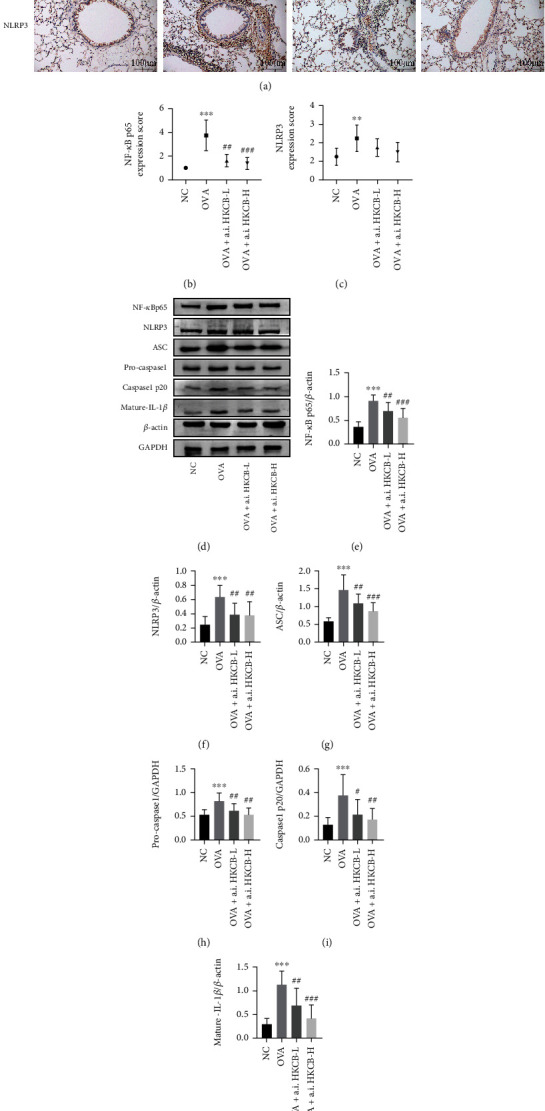
The effect of aerosol inhalation of HKCB on the NF-*κ*B/NLRP3 inflammasome signaling pathway in the lung tissues of allergic mice. (a) Expression levels of NF-*κ*B p65 and NLRP3 in lung tissues, as examined by immunohistochemistry staining (magnification 200x). (b, c) Expression scores of NF-*κ*B p65 and NLRP3 in lung tissues. (d) Expression levels of NF-*κ*B p65, NLRP3, ASC, pro-caspase1, caspase1 p20, and mature-IL-1*β* in lung tissues were examined using western blotting. (e–j) Relative expression levels of NF-*κ*B p65, NLRP3, ASC, pro-caspase1, caspase1 p20, and mature-IL-1*β* in lung tissues. ^∗∗^*P* < 0.01 and ^∗∗∗^*P* < 0.001 versus the NC group; ^#^*P* < 0.05, ^##^*P* < 0.01, and ^###^*P* < 0.001 versus the OVA group. a.i.: aerosol inhalation; CB: *Clostridium butyricum*; HKCB: heat-killed CB.

Accumulating evidence has demonstrated that activation of the NLRP3 inflammasome is involved in the development of allergic diseases, leading to its evaluation as a potential therapeutic target [22]. NF-*κ*B is a critical transcriptional regulator of the NLRP3 inflammasome [23]. To explore the effect of aerosol inhalation of HKCB on the NF-*κ*B/NLRP3 inflammasome signaling pathway, we detected the protein expression of NF-*κ*B p65 and NLRP3 in lung tissues by immunohistochemical staining. NF-*κ*B p65 and NLRP3 were predominantly expressed in the bronchial epithelial cells and inflammatory cells of the NC mice. In the lung tissues of the OVA group, NF-*κ*B p65 was upregulated in bronchial epithelial and airway inflammatory cells, and NLRP3 was mainly upregulated in airway inflammatory cells. Nonetheless, these increases were reduced in the aerosol inhalation of HKCB groups (Figures 4(a)–4(c) and Supplementary Figure S1).

Western blot analysis of lung tissues revealed that NF-*κ*B p65, NLRP3, ASC, pro-caspase1, caspase1 p20, and mature-IL-1*β* levels were significantly higher in the OVA group than in the NC group (*P* < 0.001). Meanwhile, aerosol inhalation of HKCB inhibited the expression of these proteins induced by OVA (*P* < 0.05) (Figures 4(d)–4(j)).

## 4. Discussion

In the present study, we found that aerosol inhalation of HKCB, oral HKCB administration, or oral live CB administration during sensitization could alleviate allergic airway inflammation and reduce mucus secretion in allergic mice. Moreover, aerosol inhalation of HKCB was the most effective of the three methods. Furthermore, aerosol inhalation of HKCB restored the Th1/Th2 balance, ameliorated autophagy, and inhibited the NF-*κ*B/NLRP3 inflammasome signaling pathway in the lungs of allergic mice. These results suggest that aerosol inhalation of HKCB is a promising strategy for the prevention or treatment of asthma.

The dosage of oral probiotics was mainly 10^7^-10^10^ CFU/mouse/day for 2 weeks to more than 1 month in the study of asthmatic mice [24–26]. In the present study, the mice received oral live CB CGMCC0313-1 (1 × 10^8^ CFU/mouse/day) for 2 weeks during sensitization as the oral high-dose live CB group, according to the study by Zhang et al. [14]. Moreover, we designed an oral low-dose live CB (1 × 10^7^ CFU/mouse/day) group. We found that oral live CB administration reduced airway inflammation and mucus secretion in allergic mice in a dose-dependent manner, which is consistent with the results of a previous study [25]. In the clinical trial of CB for asthma, Liao et al. found that oral administration of CB combined with antigen-specific immunotherapy can improve the clinical score and normalize the serum-specific IgE of asthmatic patients, but CB alone is ineffective [27]. In the present study, we found that oral low-dose live CB has a poor antiasthmatic effect. Insufficient dose maybe one of the reasons why CB alone was ineffective in a study by Liao et al. [27]. In addition, we found that oral HKCB CGMCC0313-1 has beneficial effects similar to those of CB CGMCC0313-1 against allergic airway inflammation. However, oral HKCB administration is safer than oral live CB administration as it carries no risk of infection.

The route of probiotic administration is an important determining factor in the development of an immune response. Airway mucosal immunity has some potential benefits such as painlessness, simplicity, low dose, good tolerance, and safety [28]. Many studies have indicated that nasal probiotic administration can efficiently regulate the immune response in several chronic respiratory diseases, including allergic rhinitis, asthma, and chronic pulmonary obstructive disease, sometimes more effective than the oral route [6, 29]. However, there are questions about the safety of nasal probiotics administration, as it can easily cause lung infection or bacteremia. Recently, Pivniouk et al. showed that airway administration of a bacterial lysate (OM-85) could protect against experimental allergic asthma via multiple immune pathways, at a dose 27–46 times lower than the reported effective oral dose [30]. In the present study, eight mice were administered atomized HKCB solution (1 × 10^7^ or 1 × 10^8^ CFU) at a time, and the quantity of HKCB inhaled into the mouse lungs was much lower than that administered in the oral HKCB group. However, aerosol inhalation of HKCB had a better protective effect against allergic airway inflammation than oral HKCB or oral live CB administration. These data suggest that aerosol inhalation of heat-killed probiotics or their components may be a promising strategy for asthma prevention or treatment.

A disturbed balance of Th1/Th2, which involves a bias towards Th2 cell activation and insufficient Th1 activation, plays a crucial role in the development of allergic inflammation [20]. Th2 cytokines, including IL-4, IL-5, and IL-13, promote inflammatory cell infiltration, goblet cell hyperplasia, and airway remodeling [31]. The Th1 cytokine IFN-*γ* inhibits Th2 cell responses [20]. Many studies have demonstrated that oral probiotics or heat-killed probiotics improve the imbalance of Th1/Th2 in allergic asthma [11, 14, 32]. In the present study, we measured the levels of IFN-*γ* and IL-4 in the BALF. We found that aerosol inhalation of HKCB during sensitization elevated the level of IFN-*γ* and markedly inhibited the level of IL-4 in the BALF. The protective effect of aerosol inhalation of HKCB may therefore be achieved through regulation of the Th1/Th2 balance.

Autophagy is a fundamental intracellular and physiological process involved in maintaining cellular homeostasis and adapting to continually changing environments. Nevertheless, abnormal autophagy has been implicated in the pathogenesis of many diseases, such as neurodegeneration, cancer, inflammation, and infectious diseases [21]. Recent evidence has demonstrated that autophagy plays an important role in the allergic airway inflammatory response and airway remodeling in asthma [33, 34]. Gong et al. showed that an oral yeast fermentate prebiotic could inhibit allergic airway inflammation by suppressing autophagy [35]. However, whether aerosol inhalation of heat-killed probiotics could inhibit autophagy in allergic airway inflammation remained unclear. In the present study, we found that enhanced autophagy of bronchial epithelial cells and airway inflammatory cells is involved in the pathogenesis of allergic asthma, which is consistent with the results of a previous study [36]. In addition, the abnormal expression of autophagy-related proteins (LC3B, Beclin1, and p62) was markedly reversed by aerosol inhalation of HKCB. This indicates that aerosol inhalation of HKCB may exert a protective effect against allergic airway inflammation by ameliorating autophagy.

NF-*κ*B is a vital driver of the inflammatory response and plays a critical role in the development of allergic airway inflammation [37]. The NLRP3 inflammasome, a downstream agent of NF-*κ*B, consists of the NLRP3 protein, ASC, and pro-caspase1 [23]. After the NLRP3 inflammasome is activated, pro-caspase1 is cleaved to caspase1, which subsequently cleaves pro-IL-1*β* and pro-IL-18 into the active proinflammatory response cytokines IL-1*β* and IL-18 [38]. Previous studies have shown that the NLRP3 inflammasome is also involved in the pathogenesis of allergic asthma [22]. Inhibition of the inflammasome activity of NLRP3 can reduce airway inflammatory cell infiltration and mucus secretion in allergic asthma [39]. In this study, the expression levels of NF-*κ*B p65, NLRP3, ASC, pro-caspase1, caspase1 p20, and mature-IL-1*β* were elevated in the lungs of OVA-induced mice. This suggests that the NF-*κ*B/NLRP3 inflammasome signaling pathway is activated in OVA-induced mice. However, aerosol inhalation of HKCB during sensitization suppressed OVA-induced activation of the NF-*κ*B/NLRP3 inflammasome signaling pathway.

## 5. Conclusions

In conclusion, our study demonstrated that aerosol inhalation of HKCB during sensitization could alleviate allergic airway inflammation by restoring the Th1/Th2 balance, ameliorating autophagy, and inhibiting the NF-*κ*B/NLRP3 inflammasome signaling pathway. These results suggest that aerosol inhalation of HKCB is a promising strategy for the prevention and treatment of asthma.

## Data Availability

The data used to support the findings of this study are available from the corresponding author upon request.

## Supplementary Materials

Supplementary Materials Figure S1: the negative control for the immunohistochemical staining, in which sterile phosphate-buffered saline was used instead of a primary antibody.
