# Supplementary material for: Aerosol Inhalation of Heat-Killed Clostridium butyricum CGMCC0313-1 Alleviates Allergic Airway Inflammation in Mice
Source: J Immunol Res. 2022 Aug 5;2022:8447603. doi: 10.1155/2022/8447603 (PMC9410851; doi:10.1155/2022/8447603)
Supplement: Supplementary Materials — Figure S1: the negative control for the immunohistochemical staining, in which sterile phosphate-buffered saline was used instead of a primary antibody. [file 8447603.f1.docx]

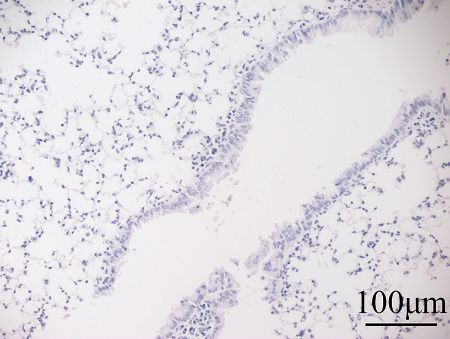

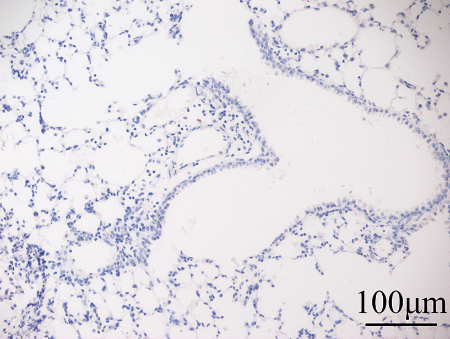


LC3B

Beclin1

p62

NF-κB p65

NLRP3


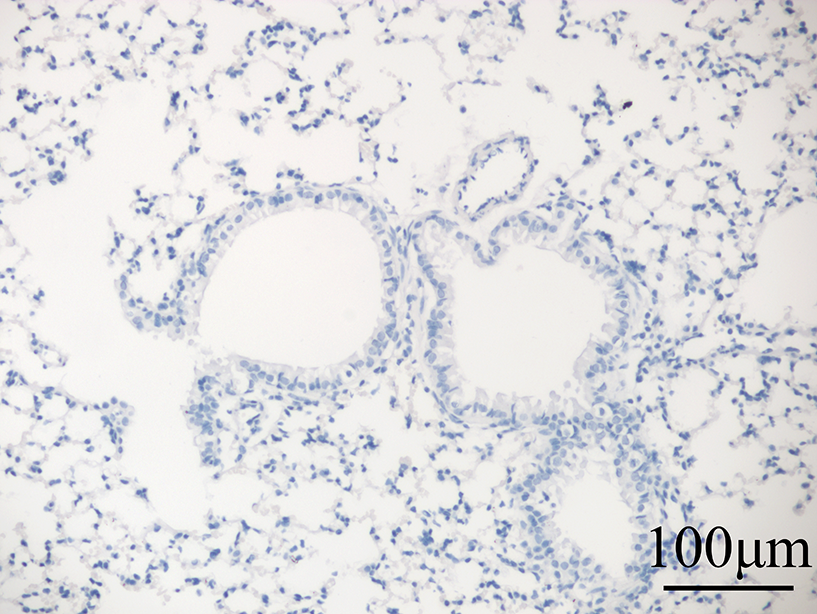

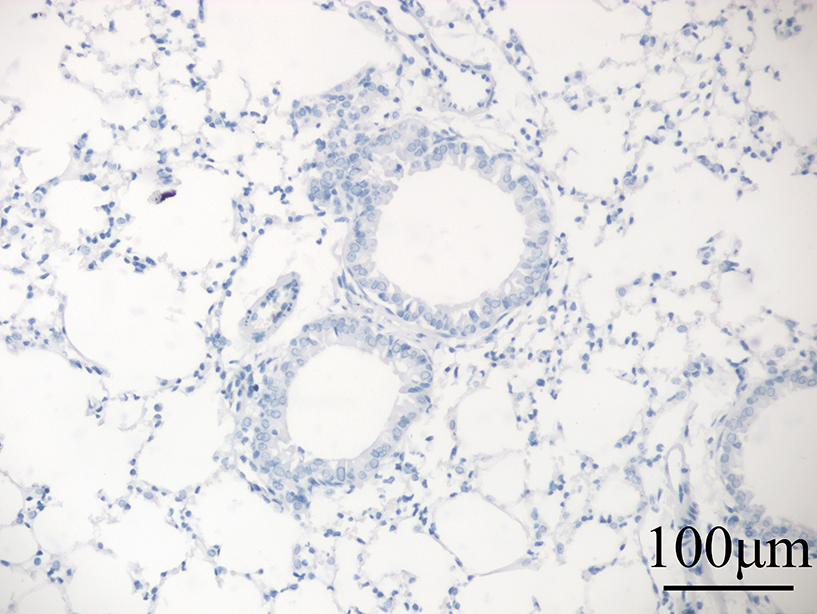

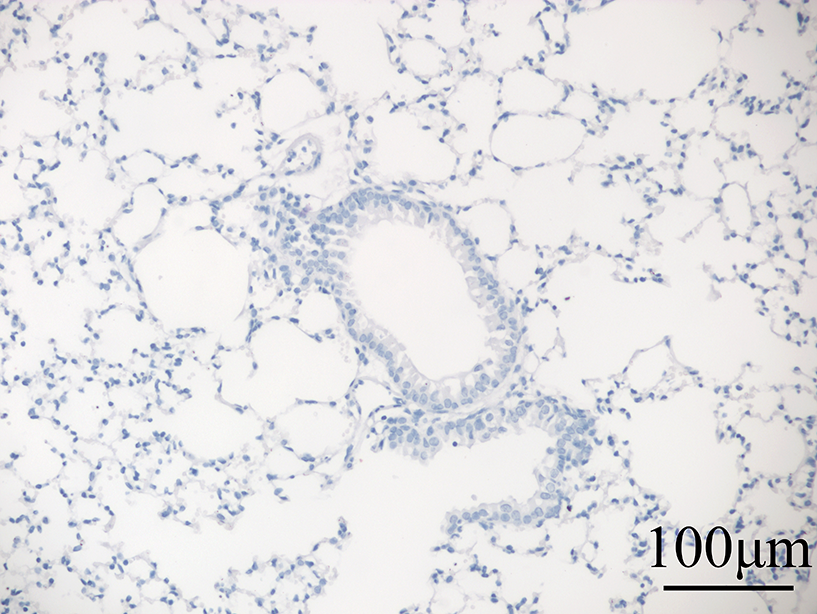


Figure S1 Negative control of immunohistochemical staining in lung tissues of mice
